# Insights into the Activation of Unfolded Protein Response Mechanism during Coronavirus Infection

**DOI:** 10.3390/cimb46050261

**Published:** 2024-05-05

**Authors:** Panagiotis Keramidas, Maria Pitou, Eleni Papachristou, Theodora Choli-Papadopoulou

**Affiliations:** Laboratory of Biochemistry, Department of Chemistry, Aristotle University of Thessaloniki, 54124 Thessaloniki, Greece; pankerdim@chem.auth.gr (P.K.); margeopit@chem.auth.gr (M.P.); epapachristou@chem.auth.gr (E.P.)

**Keywords:** unfolded protein response, IRE1, ATF6, PERK, ER stress, coronavirus, CoV, pharmacological inhibition

## Abstract

Coronaviruses represent a significant class of viruses that affect both animals and humans. Their replication cycle is strongly associated with the endoplasmic reticulum (ER), which, upon virus invasion, triggers ER stress responses. The activation of the unfolded protein response (UPR) within infected cells is performed from three transmembrane receptors, IRE1, PERK, and ATF6, and results in a reduction in protein production, a boost in the ER’s ability to fold proteins properly, and the initiation of ER-associated degradation (ERAD) to remove misfolded or unfolded proteins. However, in cases of prolonged and severe ER stress, the UPR can also instigate apoptotic cell death and inflammation. Herein, we discuss the ER-triggered host responses after coronavirus infection, as well as the pharmaceutical targeting of the UPR as a potential antiviral strategy.

## 1. Introduction

The endoplasmic reticulum (ER) is a membrane organelle that expands throughout the cytoplasm of the eukaryotic cell. The ER plays a crucial role in essential cellular functions, including protein folding, lipid and sterol synthesis, the metabolism of carbohydrates, and calcium storage [1]. Various factors such as hypoxia, glucose deprivation, acidosis, altered calcium levels, metabolic imbalances, infections, and inflammation can disrupt the ER function, mainly protein folding [2,3]. This disruption leads to changes in the ER’s capacity to mitigate the need for proper protein folding and results in the accumulation of unfolded or misfolded proteins, a condition called ER stress. ER stress triggers the activation of the adaptive or survival unfolded protein response (UPR) system to counteract this stress [4]. However, the excessive activation of the UPR mechanism (proapoptotic UPR) can lead to adverse effects for the cell, such as apoptosis [3,5].

**Figure 1:**
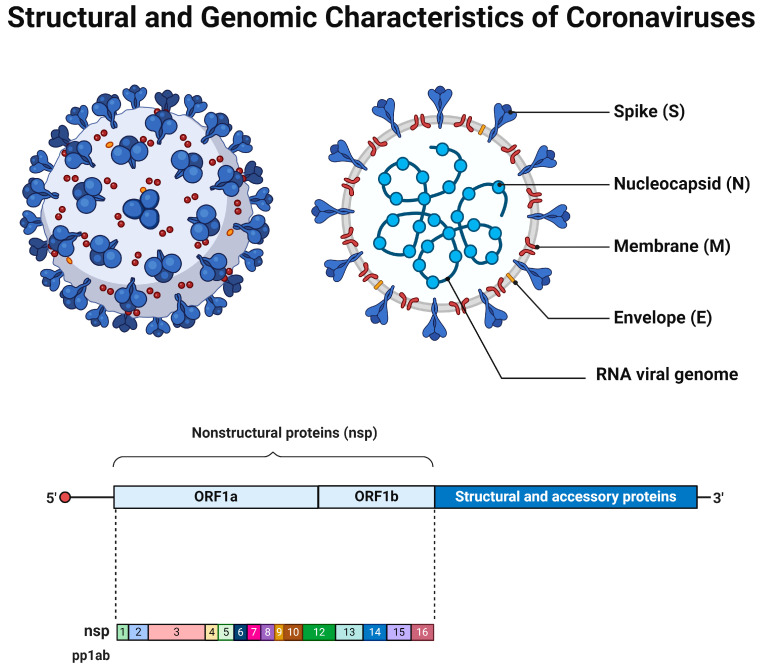
The general structural and genomic characteristics of the members of coronavirus family. Adapted from “Human Coronavirus Structure” and “Genome organization of SARS-CoV” by BioRender.com. Retrieved from https://app.biorender.com/biorender-templates (accessed on 28 March 2024).

Coronaviruses are enveloped viruses of a spherical or pleiotropic shape, with a diameter between 80 and 150 nm [6]. The International Committee on Taxonomy of Viruses has classified coronaviruses into the Order of Nidovirales, Family of Coronaviridae, and Subfamily of Orthocoronavirinae. They can be further divided into four genera: alpha (α-), beta (β-), gamma (γ-), and delta (δ-) coronaviruses [7,8]. While alpha- and betacoronaviruses primarily infect mammals, gamma- and deltacoronaviruses infect a wider range of animals, with aves being the most common host. It seems that these viruses can infect a variety of species, including humans. Notably, seven—two alpha and five beta—coronaviruses, known as the HCoV class, can infect humans and cause mild to moderate respiratory and gastrointestinal symptoms [9,10]. The additional three members (SARS-CoV, SARS-CoV-2, and MERS-CoV) have been associated with severe respiratory distress conditions, causing epidemic and/or pandemic healthcare crises [11,12,13]. In accordance with human coronaviruses, animal-infecting CoVs can cause either respiratory or enteric symptoms [14,15]. Coronaviruses possess a single-stranded positive-sense RNA molecule [(+)ssRNA] varying in size from 26 to 32 kb [16,17]. Their genomic RNA can be directly translated by infected cells to produce the structural viral proteins, spike (S), envelope (E), membrane (M), and nucleocapsid (N) proteins, that contribute to the virions’ formation and shaping. Also, it encodes the non-structural proteins (NSPs) and/or accessory proteins that enhance the virus’s replication and thus virulence [18,19,20] (Figure 1). Their genomic features appear to be critical for understanding the viral infectivity and transmission dynamics, but the natural accumulation of mutations over time due to error-prone replication, the lack of proofreading mechanisms, adaptation to the host cellular environment, and cross-species transmission result in genetic diversity regarding the coronaviruses [21,22,23].

The translation of viral proteins is strongly associated with the ER, and, upon viral infection and replication, the host cells induce a UPR [24]. For example, SARS-CoV-2 alters the structure of the ER to create replication sites, leading to ER stress and UPR activation [25]. In addition, swine coronaviruses like porcine epidemic diarrhea virus (PEDV) and transmissible gastroenteritis virus (TGEV) can induce ER rearrangement and provoke ER stress [26]. Herein, we summarize the latest data concerning the crosstalk between coronavirus infection and UPR signaling, as well as the potential of therapeutically targeting the UPR against coronavirus infections. 

## 2. The Unfolded Protein Response

The role of the UPR is to counteract the ER stress, and it serves three main purposes: adaptive response, feedback control, and cell fate. In its adaptive role, the UPR seeks to alleviate endoplasmic reticulum ER stress and restore the ER equilibrium [27]. Upon the successful mitigation of stress, feedback mechanisms deactivate the UPR signaling pathways [28]. During the adaptive response, it retains proteostasis by reducing protein synthesis, increasing membrane lipid biosynthesis and ER membrane activity, and inducing the expression of ER-luminal chaperones and the components of the ER-associated degradation machinery (ERAD) [29]. Also, the ER export is regulated, and, currently, studies have proven that it is contingent upon the condition of the folding machinery and the internal environment within the stressed ER [30]. Nevertheless, the prolonged and excessive activation of the UPR may eventually induce cell death through apoptosis and autophagy [31]. 

The crucial role of the UPR is obvious not only in health but also in diseases. The malfunctioning of the UPR has been associated with diverse diseases including cancer [32], cardiovascular diseases [33], and neurogenerative diseases like Alzheimer’s disease [34]. Furthermore, the growing evidence regarding the role of the UPR in the immunological response supports its implication in infections and inflammatory and autoimmune diseases [35]. 

During viral infections, the UPR is used as machinery to defend the host cell. However, many viruses like Zika virus, coronaviruses such as SARS-CoV-2 [36], and herpesviruses [37] have managed to hijack this system and induce massive protein expression and viral replication [38]. Positive-stranded RNA viruses like SARS-CoV-2 also recruit the UPR to induce ER membrane rearrangements and to favor the synthesis of the viral membrane [36].

### 2.1. Activation of the UPR Sensors and Signal Transduction

The UPR signals through three different transmembrane receptors, inositol-requiring enzyme 1 (IRE1), protein kinase R (PKR)-like ER kinase (PERK), and activating transcription factor 6 (ATF6). These receptors consist of three domains, the luminal, the transmembrane, and the cytosolic domain. They act as sensors by detecting the unfolded protein levels through their luminal domains and convey this information to cytosolic effector pathways through their respective cytoplasmic domains, resulting in the downregulation of protein translation and increased removal of misfolded or unfolded proteins [39,40]. 

#### 2.1.1. The IRE1 Signaling

IRE1 and PERK exhibit common activation ways. The receptors can directly be activated upon interaction with the misfolded proteins or upon dissociation from immunoglobulin-binding protein (BiP), otherwise known as glucose-regulated protein 78 (Grp78) [41]. IRE1’s structure is highly conserved through species, and two isoforms exist: IRE1a and IRE1b [42]. IRE1β functions as a dominant-negative inhibitor of IRE1a in different cell types like epithelial cells, while IREa is the main signal transducer [43]. 

The direct activation model proposes that unfolded proteins bind to a hydrophobic groove located in the N-terminal domain and permits the oligomerization and autophosphorylation of the IRE1 receptors [44]. However, the main activation pathway is through BiP, a chaperonin that holds a dual and crucial role in the ER function. In normal conditions, BiP is bound via the ERdj4/DNAJB9 into the luminal domain of IRE1 and supresses the activation of the UPR. Upon the accumulation of misfolded or unfolded proteins, the latter bind to BiP, disassociating from the receptor and enabling trans-autophosphorylation and oligomerization [40]. 

After the oligomerization of more than four monomers, the active C-terminal domain displays dual activity as it encompasses a kinase domain and an endoribonuclease domain. The kinase domain binds ATP and transphosphorylates the other monomers so as to activate the RNAase domain. This splices an intron of the x-box binding protein 1 XBP1 mRNA non-canonically, which then acts as a transcriptional factor and promotes the viability, expansion, and differentiation of cells [45,46]. The main target genes of XBP1s are the ER chaperones (*Dnajb9*, *Dnajb11*, *Pdia3*, and *Dnajc3*), the ERAD components (*Edem1*, *Herpud1*, and *Hrd1*), the folding enzymes, and the ER translocon (*Sec61a1*). Also, cell-specific genes are expressed [47]. The IRE-Xbp1 pathway is significant in various diseases, like metabolic conditions involving glucose and lipid metabolism, tumorigenesis, and cancer metastasis [48]. 

**Figure 2:**
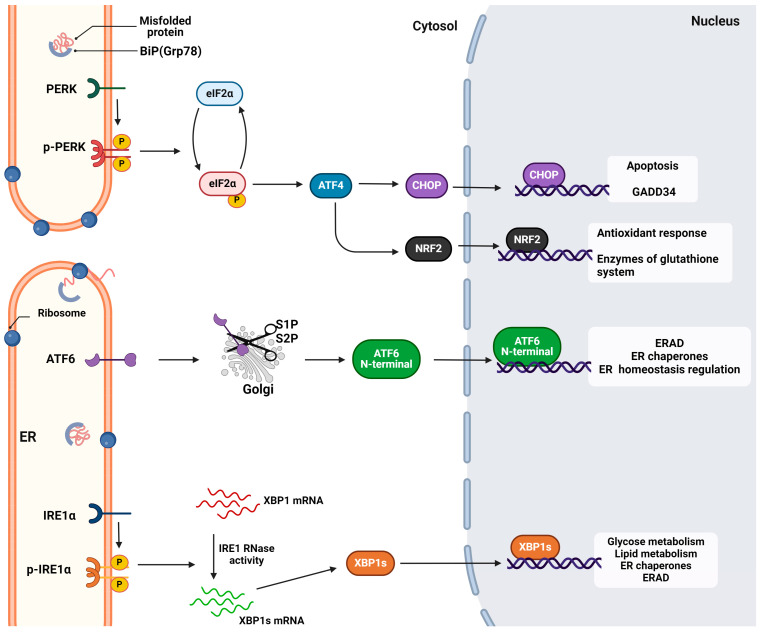
The unfolded protein response mechanism. The activation and the signal transduction of the three (ATF6, IRE1, and PERK) sensors of UPR. Each respective pathway activates the transcription of a specific pool of genes to relieve ER stress and maintain ER and cell homeostasis. However, extended activation of UPR can lead to apoptosis or other pathological conditions. Retrieved from https://app.biorender.com/biorender-templates (accessed on 28 March 2024). Adapted from “Intracellular Layout—Endoplasmic Reticulum Signaling to Nucleus” by BioRender.com (accessed on 24 April 2024).

IRE1 also interacts with TRAF2 to initiate the inflammatory response and activate the protein kinases associated with cellular apoptosis, particularly apoptosis signal-regulating kinase 1 (ASK1). This activation subsequently triggers the activation of c-Jun N-terminal kinase (JNK) [2]. In severe stress conditions, the active RNAse activates the regulated IRE1-Dependent Decay RIDD branch. This cleaves mRNAs that encode mainly ER proteins and secondly cytosolic and nucleus-related proteins, leading to cell death [49] (Figure 2).

#### 2.1.2. The PERK Signaling

The PERK branch is the most significant pathway for the host antiviral response. PERK shares a common activation pathway with IRE1 as its activation relies mainly upon the disassociation of BiP. In response to misfolded proteins, BiP is released and PERK multimerizes and is trans-autophosphorylated. The phosphorylated cytosolic domain acts as a kinase and phosphorylates the alpha subunit of the eukaryotic initiation factor eIF2 (eIF2a) specifically at Ser51. The phosphorylated eIF2a induces the expression of the bZip transcription factor 4 (ATF4), which regulates redox equilibrium, amino acid metabolism, and autophagy [50]. 

In the proapoptotic UPR, ATF4 interacts and subsequently activates CCAAT/enhancer binding homologous protein (CHOP) [51]. CHOP acts as a proapoptotic factor and upregulates the expression of growth arrest and DNA damage-inducible protein (GADD34), which plays a role in the dephosphorylation of elF2a, facilitating stress recovery. However, CHOP can also act as a pro-apoptotic factor by triggering caspase 8 via the death receptor 5 [5]. Also, the phosphorylated eIF2a suppresses the transcription of most mRNAs by inhibiting the exchange of GDP with GTP in the preinitiation translation complex. Noteworthily, eIF2a increases the transcription of ATF4 to promote cell death [3]. 

Except the regulation of cell survival and death, PERK has been linked with the direct activation of nuclear factor erythroid 2-related factor 2 (NRF2), a transcription factor implicated in the oxidative response [52]. This factor regulates the expression of the genes responsible for the glutathione system and ROS elimination. Indirectly, as reported by Sarcinelli et al., PERK upregulates ATF4 and the latter upregulates the Nrf2 transcript [53] (Figure 2).

#### 2.1.3. The ATF6 Signaling

ATF6 acts as a pro-survival branch, and its activation relies on the dissociation of BiP and exposure of the Golgi-targeting sequence. Thus, the receptor is translocated to the Golgi apparatus, and, after cleavage at sites 1 and 2, the active ATF6(N) fragment moves to the nucleus and binds to the ER stress response element (ERSE) I or II [54,55]. As a transcription factor, it upregulates the genes that encode ER chaperones Grp78 and Grp94, as well as ΧΒP1 [28]. Also, it has been reported that, except proteotoxic stress, ATF6 activation and translocation to the nucleus are mediated by the biosynthetic molecules, dihydrosphingosine and dihydroceramide, which bind directly to sequence motif VXXFIXXNY of the luminal domain [56]. 

The ATF6 branch is crucial for the retention of organelle homeostasis and ER capacity and in physiological development; it regulates osteogenesis, chondrogenesis, and neurogenesis. Its role has also been reported in diseases like cardiac hypertrophy [57] and ischemia [58]. The role of the ATF6 branch has also been investigated in viral infections and reported various responses [59] (Figure 2).

### 2.2. UPR Crosstalk with Other Pathways

The UPR is closely related to ERAD. This machinery comprises three stages: the recognition, reverse translocation, and degradation of misfolded proteins. During the selective recognition of substrates from chaperonins, the primary obstacle for the ERAD system is differentiating misfolded proteins from properly folded ones, a task made more difficult by the wide variety of secretory proteins [60]. After recognition, the proteins are reversely translocated through translocation channels, like Sec61, ERAD-L, and ERAD-M [61]. In this process, PERK and IRE1 crosstalk, and, specifically, PERK exerts an activation action on recognition, translocation, and degradation, while IRE1 impacts the translocation through the ER components and translocon upregulation [62].

ER stress has been extensively studied as a trigger of autophagy, a cellular process responsible for the degradation of malfunctioning organelles or protein aggregates. In this process, the three branches of the UPR cooperate and induce the formation of autophagosomes. However, the PERK--eIF2α-ATF4-CHOP cascade has a significant role in the expression of the ATG and LC3 genes [60,63]. Also, the IRE1a-XBP1 axis triggers the expression of Beclin-1, a protein that initiates autophagy [64]. A specific type of autophagy for the destruction of damaged mitochondria, mitophagy, has been reported to be initiated via the activation of ATF4 [65].

There is an interconnection between the signaling pathways of UPR, autophagy and oxidative stress response. Normally, the ER during protein synthesis produces excess amounts of reactive oxygen species (ROS), and its redox state is closely controlled [52]. To maintain redox homeostasis, a closed loop is triggered. PERK forms a complex with endoplasmic reticulum oxidase 1 (ERO1a), a protein activated from the PERK--eIF2α-ATF4-CHOP axis. ERO1a is responsible for the oxidation of reduced PDI and consequently disulfide bond formation [66].

## 3. Coronaviruses and ER Stress

As previously mentioned, the interaction of coronavirus infection and the UPR has been extensively reported. This section of the review will cover examples of coronaviruses from each genus that activate the UPR and how they manipulate each UPR transducer to promote infection.

### 3.1. Alphacoronaviruses: The Cases of Porcine Enteric Coronaviruses

#### 3.1.1. Porcine Epidemic Diarrhea Virus (PEDV)

Porcine Epidemic Diarrhea Virus (PEDV) is a member of the alphacoronaviruses subfamily [67], which primarily infects swine and is characterized by high mortality rates, especially in piglets. PEDV targets cells from the small intestine, causing symptoms such as diarrhea, dehydration, and vomiting. It is a pleiomorphic enveloped virus, whose virion possesses the characteristic crown-like structure of coronaviruses [68]. From a genomic perspective, PEDV contains a 28 Kb (+)ssRNA, which encodes sixteen non-structural proteins, four structural (S, E, M, and N), and one accessory protein (ORF3). Although it appears to emerge in epidemic outbreaks, the last decade, due to its high mutability, resulted in the global outbreak of more severe strains that negatively affect the swine industry [69]. Here, the latest insights regarding the importance of the UPR mechanism in PEDV infections are summarized. 

The PEDV infection of Vero cells induced ROS generation, which in turn activated all three UPR signaling pathways. Furthermore, it was demonstrated that IRE1 and PERK, but not ATF6, induce ER stress-dependent autophagy, a procedure that promotes PEDV replication [70]. PEDV infection can induce PERK activation and eIF2a phosphorylation to upregulate ERO1—an essential factor for oxidative folding. ERO1a overexpression leads to ROS accumulation and increased viral titer, indicating oxidative stress as an important feature for facilitating viral infection [71]. Apart from the main UPR transducers, PEDV infection can cause the elevated expression of UPR executioners such as HERPUD1, GRP78, and CHOP [72]. Moreover, the activation of the UPR was also reported in vivo. In particular, Chen et al. reported an upregulation of ATF4, Grp78, and the activated forms of ATF6 and IRE1 in jejunal epithelial cells isolated from PEDV-infected weaned pigs. The extended activation of the UPR eventually resulted in the cleavage of caspase 3 and thus in ER-induced apoptosis [73].

**Figure 3:**
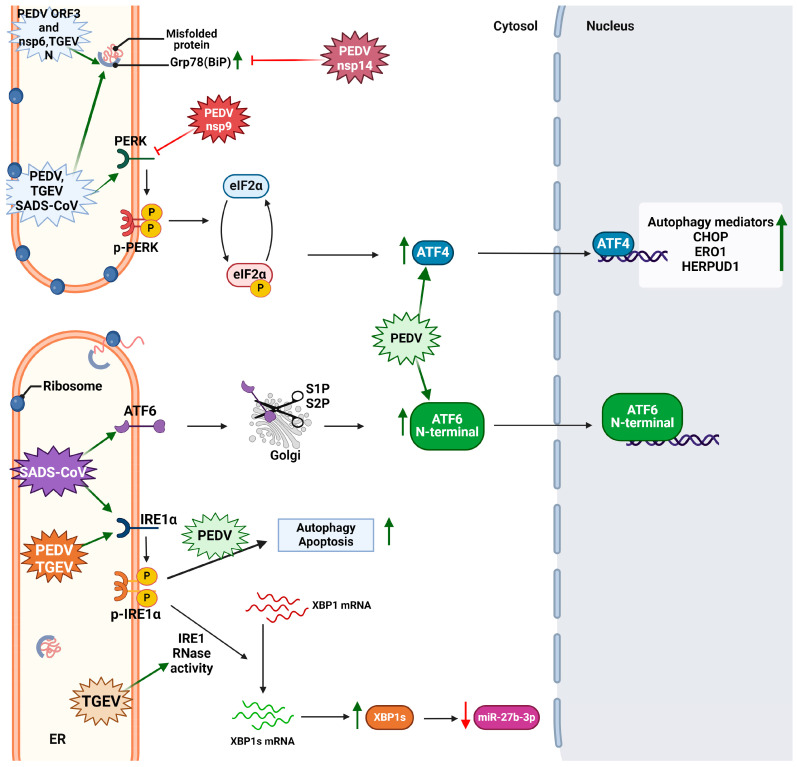
The interference of alphacoronaviruses and their viral factors on various UPR elements. The green arrows indicate positive regulation of the respective factor. Retrieved from https://app.biorender.com/biorender-templates (accessed on 28 March 2024). Adapted from “Intracellular Layout—Endoplasmic Reticulum Signaling to Nucleus” by BioRender.com (accessed on 24 April 2024).

Recent reports also indicate the potential role of the PEDV proteins in ER stress and UPR activation. For instance, the overexpression of PEDV nsp6 in Marc145 cells can activate the ER stress-induced apoptotic pathways of IRE1 and PERK, but not those of ATF6, to degrade GRAMD4, a host factor that was demonstrated to inhibit virus replication [74]. PEDV Nsp9 interacts with H2BE, causing H2BE overexpression by inhibiting IRX1, a regulator of H2BE expression. H2BE overexpression inhibits the PDEV-induced upregulation of PERK, IRE1, and their respective apoptotic executioners, thus suppressing cell death and contributing to viral replication [75]. PEDV nsp14, via its N7-TMase domain, can suppress Grp78 expression. More precisely, nsp14 can bind to the Grp78 promoter, inhibiting its expression and ER stress and thus promoting PEDV replication [76]. The ORF3 protein is also highlighted to possess an infectious role during PEDV infection. ORF3 resides at the endoplasmic reticulum of transfected cells, triggering ER stress by upregulating Grp78 and activating the PERK-eIF2a pathway of the UPR, which in turn induces autophagy but not apoptosis [77]. The sites that PEDV and its proteins intervene in regarding the UPR mechanism are shown in Figure 3. The high mutation rate of PEDV and its impact on the swine industry make it necessary to deepen our knowledge regarding the virus–host interaction, as well as the contribution of each protein to viral pathogenesis.

#### 3.1.2. Transmissible Gastroenteritis Virus (TGEV)

Another member of the alphacoronavirus subfamily, closely related to PEDV, is TGEV. It is one of the major infectious agents responsible for the enteritis and severe diarrhea observed in swine, with its mortality rates reaching 100% in the piglet population [78]. As a coronavirus, it contains a single-stranded genomic RNA with a size of 28.5 Kb approximately, encoding sixteen NSPs, four structural (S, M, N, and E), and three accessory (ORF3a, ORF3b, and ORF7) proteins [79]. Most of the time, TGEV infection is confused with PEDV as both cause GI symptoms and show similar clinical characteristics. It is reported that both PEDV and TGEV can be present as a mixed infection at the same time [80]. In this review, we outline the importance of ER stress and UPR activation for TGEV virulence.

TGEV infection has been reported to induce ER stress both in vitro and in vivo. The dual role of PERK-eIF2a phosphorylation in terms of repressing viral replication has been further elucidated. In particular, the activation of this UPR axis can lead to a translational shut off for both host and viral proteins as well as in nFκΒ activation and IFN-I production. Both of these events lead to the attenuation of TGEV replication and infection [81]. Furthermore, miR-27b demonstrated to inhibit the TGEV replication both in vitro and in vivo. TGEV infection induces ER stress and activates all three branches of the UPR, but only the IRE1 pathway is found to be responsible for the regulation of miR-27b expression. Particularly, during TGEV infection, the activated IRE1 functions as an RNase, leading to the overexpression of the XPB1s, a transcription factor that inhibits miR-27b-3p production and thus promotes virus survival [82]. TGEV proteins can also trigger the UPR. nsp1 residues 85–102 were found to be responsible for the PERK activation and eIF2 phosphorylation in IPEC-J2 cells, resulting in nFκΒ activation and thus interferon lambda production (IFN-λ1 and IFN-λ3). According to Zhang et al., the TGEV nucleocapsid protein is localized in cytosol and is capable of inducing ER stress. Grp78—an ER stress marker—was upregulated both in mRNA and at the protein level in cells transfected with TGEV nucleocapsid protein [83]. The sites of UPRs that are perturbated by TGEV and its proteins are illustrated in Figure 3.

#### 3.1.3. Swine Acute Diarrhea Syndrome (SADS-CoV) 

SADS-CoV is the newest member of alphacoronaviruses, detected for the first time in China in 2017. Although it was under control, it emerged again in 2019 [84]. Its genomic RNA can be translated into 15–16 nsp, four structural, and four accessory (ORF3, ORF6, ORF7, and hemagglutinin esterase) proteins. SARS-CoV-2 occurrence and pandemic outbreak led scientists to closely monitor other coronaviruses as well due to the high probability for cross-species transmission and severe cases of infection [26]. Recently, it was indicated that SADS-CoV upregulates Grp78, thus causing ER stress, and induces all three branches of the UPR both in vitro and in vivo. SADS-CoV and its papain-like protease can recruit the IRE1 pathway of the UPR to promote autophagy-induced viral replication [85] (Figure 3). However, our knowledge regarding SADS-CoV interactions with the host’s cells is limited due to the short time that has passed since its first appearance.

### 3.2. Betacoronaviruses: The Case of SARS-CoV-2

SARS-CoV-2 is the most recent addition to the betacoronavirus genus and the infectious agent of the COVID-19 disease. Its structural and genomic characteristics are inconsistent with the other members of the coronavirus family, being a spherical virus with a diameter of almost 125 nm and having the characteristic spike projections on its surface, while at the same time possessing a single-stranded sense RNA [(+)ssRNA] of about 30 kb [86,87]. After the viral entry to host cells through the ACE2 receptor, the genomic RNA is released into the cytoplasm to be translated into four structural proteins (S, M, E, and N), sixteen NSPs, and nine accessory proteins, all of which contribute to viral replication and pathogenicity. A common mechanism, which seems to be recruited to facilitate the viral infection, is the unfolded protein response (UPR) [88]. An extensive analysis of whether and how SARS-CoV-2 activates and regulates the UPR pathways is provided below.

Early studies regarding the correlation between SARS-CoV-2 infection and the UPR highlighted that, although the SARS-CoV-2 infection activates the ER stress signaling at the mRNA level, it suppresses the protein expression of the essential ER stress-related factors such as Grp78, IRE1, and HERPUD1. After an in-depth proteomic analysis, it was found that thapsigargin, a UPR activator, exhibits a potent antiviral effect across different cell types as it regulates the pathways and proteins involved in the ER quality control (ERQC) or ERAD pathways that are otherwise inhibited by the viral infection [89]. However, later studies counteracted this argument as it was reported that the SARS-CoV-2 infection of the Vero-E6-ACE2 and H1299 cells at an MOI = 0.5 resulted in the overexpression of Grp78 not only at the mRNA level but at the protein level at 36 h following infection. Also, Shin et al. observed reduced viral replication when Grp78 was inhibited, indicating the significance of ER stress regarding the SARS-CoV-2 pathogenesis [90]. The importance of Grp78 and its upregulation during COVID-19 was further indicated in vivo as its expression was found to be abundant in autopsied lung tissues from COVID-19 patients [91], as well as in hamster lung tissues and human lung organoids infected with SARS-CoV-2 [92]. These findings were also consistent with the protein–protein interaction (PPI) network analysis from Sinha et al., who highlighted that ER stress is a major response for the emergence of post-COVID-19 lung disease [92]. Moreover, in HCAEC endothelial cells, the EDEM1 mRNA—an ER stress-induced factor that disposes of misfolded proteins—was upregulated 24 and 48 h following infection, but not in Grp78, CHOP, and ATF4, indicating differences regarding the ER stress response mechanism during the virus infection [93]. Furthermore, it was demonstrated that SARS-CoV-2 infection can regulate the UPR activation at the level of miRNAs. Particularly, an analysis in blood samples collected following COVID-19 demonstrated that SARS-CoV-2 infection significantly downregulates or alters the expression of a series of miRNAs, such as miR-355-5p, which are related to ER stress activation or UPR sensors [94].

**Figure 4:**
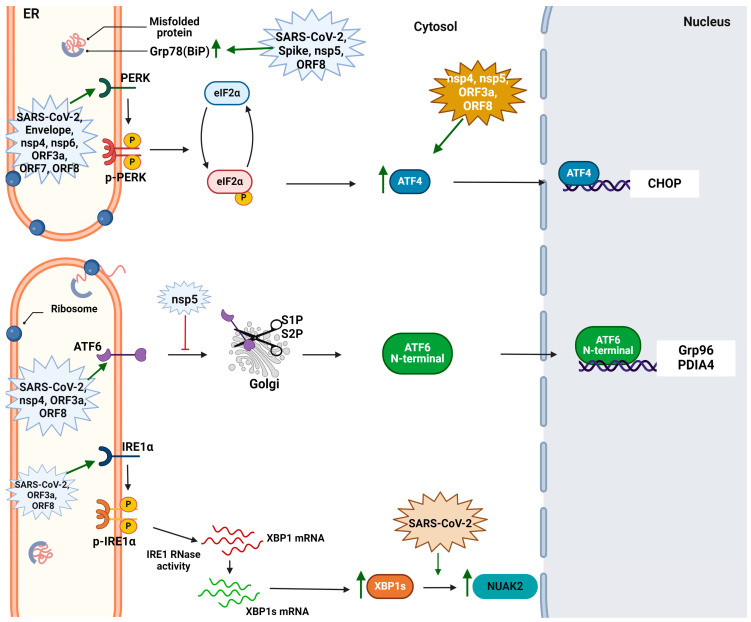
The perturbation of SARS-CoV-2 and its viral factors on the three pathways of UPR mechanism. The green arrows indicate positive regulation of the respective factor. Retrieved from https://app.biorender.com/biorender-templates (accessed on 28 March 2024). Adapted from “Intracellular Layout—Endoplasmic Reticulum Signaling to Nucleus” by BioRender.com (accessed on 24 April 2024).

However, an intriguing question that arises is why the virus needs the mechanism of the UPR and how it recruits its individual transducers. The SARS-CoV-2 infection of Vero cells causes ER stress and triggers the activation of all three branches of the UPR, which in turn favors viral replication [95]. Nguyen et al. demonstrated that, although SARS-CoV-2 induces IRE1a activation by autophosphorylation, it inhibits its RNase activity in A549- and Calu-3-infected cells. The absence of the IRE1 RNase activity restrains the expression of the downstream IRE1a/XBP1s UPR executioners, enabling the virus to hijack the host immune system [96]. Indeed, the IRE1 overexpression in the Vero-infected cells induced the upregulation of cleaved caspase-9 as well as nFκΒ activation and TNF-a expression, which were ameliorated when the cells were treated with the antiviral agent Nelfinavir [97]. Although the findings from Oda et al. in HCT-9, Calu-3, and Vero-3 indicated the activation of the UPR and IRE1a pathway, they contradict previous reports as they found in samples from patients with severe COVID-19 the overexpression of both activated IRE1a and XBP1s, thus indicating the RNase activity of IRE1 [98]. Nevertheless, all the reports indicate the significance of the IRE1a branch in SARS-CoV-2 infection. Furthermore, it was displayed that SARS-CoV-2 infection leads to NUAK2 upregulation, an executioner kinase of the IRE1a-XBP1 pathway, which was found to be crucial for viral replication as it promotes cell surface ACE2 abundance and therefore the SARS-CoV-2 entry into the host cells [25]. Also, there are some early indications that SARS-CoV-2-induced ER stress triggers gastrointestinal symptoms. More specifically, SARS-CoV-2 exerts stress into the endoplasmic reticulum of enterocytes, resulting in the production of DAMPs. These released DAMPs increase the expression and release of vasoactive intestinal peptide (VIP) by enteric neurons, thus affecting the fluid absorption in the enterocytes and causing diarrhea symptoms [99]. In Figure 4, the interference of SARS-CoV-2 with the UPR mechanism is indicated.

All the reports mentioned above indicate the correlation of SARS-CoV-2 with ER stress and the UPR mechanism, as well as the importance of the UPR in COVID-19 complications. However, a more comprehensive perspective on how this machinery is recruited during the viral infection is provided by experiments involving individual proteins: structural, NSPs, and accessory proteins. For example, in human bronchial epithelial cells, the spike protein interacts with the host factors related to ER stress and the unfolded protein response, such as Grp78, Calnexin, thrombospondin-1, and FBX-2. It was further reported that the D614 spike protein shows greater affinity to Grp78 and Calnexin than the G614 mutant [100]. In accordance, the envelope (E) protein of SARS-CoV-2 shows the potential to switch on the UPR mechanism. The transient transfection of HEK293T cells with the SARS-CoV-2 E protein activated the PERK branch of the UPR and induced the phosphorylation of eIF2a. Additionally, the site mutations (N15A or V25F) in the E protein, which are essential for its oligomerization, do not affect the phospho-eIF2a levels and thus PERK activation [101]. The implications of the structural proteins in the UPR mechanism are indicated in Figure 4. The reports regarding the interference of the structural proteins of SARS-CoV-2 in the UPR mechanism are quite limited; thus, further research on the interaction of these proteins with the UPR mechanism and its activation is required. 

Apart from the involvement of the structural proteins in the UPR, there are indications of non-structural proteins (NSPs) inducing ER stress and UPR transducers, such as that of nsp3. The N-terminal of nsp3 (nsp3.1) demonstrated to interact with ATF6, suppressing its activation and the mRNA and protein levels of its pathway executioners, GRP94 and PDIA4. However, nsp3.1 expression does not affect the regulation of PERK and IRE1 [102]. In accordance with Davies et al., the nsp4 protein induces both the PERK-ATF4-CHOP and ATF6 pathways, but the latter to a more moderate extent. The authors further validated these findings by quantifying the proteome variations using tandem mass tag LC-MS/MS both in HEK293T and A549 cells transfected with nsp4 [103]. Furthermore, the potential role of the Main protease (Mpro or nsp5) in the emergence of gastrointestinal symptoms during SARS-CoV-2 infection was suggested. The transfection of HCT116 cells with Mpro resulted in CHOP and Grp78 upregulation, and thus ER stress, only when LCAT3 cleavage was observed. Thus, GI symptoms can be possibly associated with Mpro-induced LCAT3—an intestinal lysophospholipid acyltransferase important for lipid absorption—cleavage [104]. As with the previously mentioned nsps, nsp6 appears to be related to ER stress induction. NSP6 is an ER-residing viral protein that suppresses IFN production by triggering PERK-specific induced autophagy and STING1 degradation. More specifically, Jiao et al. reported that residues 81–120 are essential for hijacking the ER stress-induced immune response. It appears that several structural and non-structural proteins can sufficiently induce ER stress and activate the UPR mechanism to facilitate the infection and evade the host immune system [105] (Figure 4). However, it remains unclear whether the other members of non-structural proteins can potentially activate these cellular events.

Among the most studied accessory proteins for its implications in the mechanism of the UPR is ORF3a. Although earlier studies indicated that ORF3a could not upregulate the UPR, as spike and ORF8 could [95], a strong correlation between the ORF3a and ER procedures was later suggested. ORF3a activates all three branches of the UPR and recruits the IRE1 and ATF6 pathways to induce incomplete autophagy in a FIP200/Beclin-1-dependent manner, indicating the important crosstalk between the ORF3a-induced UPR and the canonical pathway of autophagy [106]. Furthermore, Keramidas et al. elucidated the role of the PERK branch in A549 cells transfected with ORF3a mRNA. In particular, ORF3a functions as an ER stressor that triggers terminal UPR, inducing apoptosis and the nFκΒ-dependent inflammatory response through the activation of the PERK-ATF4-CHOP pathway [107]. The close interaction between ORF3a and ER stress was further indicated by Zhang et al. ORF3a induces ER-phagy through its interaction with the HMGB1-BECN1 complex. This induction of reticulophagy results in ER stress and thus in a proinflammatory response and ER-dependent apoptosis [108]. ORF3a can also interact with factors related to ER lumen, such as CLCC1, and eventually trigger the UPR, indicating that such interactions may play a role in SARS-CoV-2-induced cytotoxicity [109]. A more recent study from Zhang et al. pointed out that the localization of non-natural occurring ORF3a mutants can affect the outcome of UPR activation. For instance, lysosomal ORF3as can induce the IRE1-XBP1s pathway of the UPR and autophagy more efficiently compared to ER-residing ORF3as. However, the ER-ORF3as, although they are eventually degraded by the ERAD mechanism, demonstrate more intense cytopathic responses via the ER stress–reticulophagy response [110]. Moreover, there are suggestions that some of the other accessory proteins can interact with the UPR mechanism too. ORF7a interacts via two lysine residues of the C-terminal region with BclXL, recruiting it to the ER and activating the PERK-CHOP cascade and thus apoptosis. However, during an infection, ORF7a seems to recruit, at the same time, the ubiquitination mechanism to hijack the cell defense mechanisms and ameliorate its interaction with BclXL, thus weakening the ER stress activation and favoring cell survival and virus replication [111]. The ORF8 of SARS-CoV-2 was characterized as an N-glycosylated protein, which resides in the ER and interacts with Grp78 and Calnexin, two ER stress-related factors. The ORF8 overexpression in HEK293T cells upregulates the IRE1-XBP1 pathway of the UPR, as well as CHOP and Grp78, in a calnexin-expression dependent manner [112]. Furthermore, ORF8 can form complexes with ER proteins via disulfide bridges, thus escaping its degradation. This mechanism of action leads to an ORF8-induced adaptive UPR either through ORF8 direct binding to IRE1a and PERK or by competing for the Grp78 binding to the UPR sensors [113]. Sheng et al., utilizing omics analysis, particularly an integration of transcriptome and interactome data, verified the involvement of the ORF8 protein in the biological processes associated with the endoplasmic reticulum. This is further confirmed by the physical interaction between ORF8 and SERPINE1, a factor that, when overexpressed, restrains ORF8-induced ER stress to promote viral replication [114]. Similar behavior is also shown by the two most circulated SARS-CoV-2 ORF8 genotypes, orf8L (L84) and orf8S (S84). Both ORF8L and ORF8S could induce ER stress in HEK293T cells by overexpressing the GRP78 and GRP96 stress markers and subsequently inducing the activation of two out of the three UPR branches—the ATF6 and IRE1 pathways. Although both genotypes could antagonize the IFN beta expression, it remains unclear if this inhibition is due to UPR activation [115]. ORF8 was also suggested as a factor that is crucial for SARS-CoV-2 pathogenicity. In particular, ORF8 physically interacts and forms condensates with p62, leading to ER stress and ER-phagy inhibition. Furthermore, these aggregates were found to prevent the ER-phagic degradation of viral double membrane vesicles (DMVs), which are essential for viral replication [116]. Hence, studies involving accessory proteins reveal that there is a high correlation of SARS-CoV-2 infection with alterations in ER physiology and thus the induction of stress on it. The involvement of accessory proteins in the UPR mechanism is illustrated in Figure 4. Nevertheless, the role of the other ORF proteins, such as ORF6, ORF9, and ORF10, as well as novel interactions that may ultimately activate these UPR mechanisms and contribute to viral pathogenesis, need to be further confirmed.

### 3.3. Gammacoronaviruses: The Case of Infectious Bronchitis Virus (IBV)

Infectious Bronchitis Virus (IBV) is the first-ever characterized coronavirus and the standard model of gammacoronaviruses. It is spread worldwide among chicken populations of all ages and causes a highly contagious disease that affects multiple tissues, such as the lungs, kidneys, and reproductive organs. IBV is an enveloped virus with a genomic size of 27.6 Kb. Its genomic RNA is translated into fourteen non-structural proteins, four structural proteins, and four accessory proteins [117,118]. Infectious bronchitis, due to the rapid IBV mutations, can now be diagnosed in different avian species beyond chickens, leading to constant—health and economic—risk factors for the poultry industry [119]. As the first known coronavirus and study model for gammacoronaviruses, it is known that IBV can recruit ER stress and the UPR mechanism to promote viral pathogenesis [120]. Hence, in this review, we will present the latest studies that indicate UPR activation during IBV infection. 

**Figure 5:**
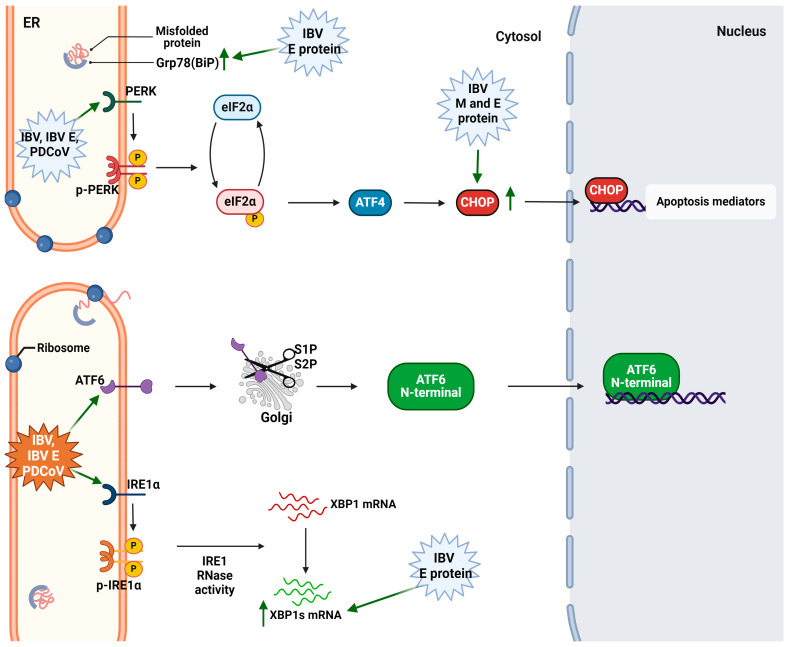
The interference sites of IBV (gammacoronavirus) and PDCoV (deltacoronavirus) in UPR mechanism. The green arrows indicate positive regulation of the respective factor. Retrieved from https://app.biorender.com/biorender-templates (accessed on 28 March 2024). Adapted from “Intracellular Layout—Endoplasmic Reticulum Signaling to Nucleus” by BioRender.com (accessed on 24 April 2024).

IBV infection induces ER stress, activating all three branches of the UPR, but, among the three sensors (IRE1, ATF6, and PERK), only IRE1 was found to be required for the modulation of autophagy [121]. Another study demonstrated the role of the PERK pathway in IBV-infected cells. In particular, PERK activation and eIF2a phosphorylation promote the transcriptional upregulation of IL-8, thus indicating a possible induction of the inflammatory response [122]. Apart from the whole virus, its individual proteins were found to contribute to the UPR activation. The membrane (M) protein of the IBV was characterized as a glycosylated protein, and the glycosylation in the N-terminal ectodomain was found to contribute to the ER stress induction as Liang et al. reported considering the elevated expression of the apoptotic (CHOP and PARP-1) and proinflammatory (IL-6 and IL-8) biomarkers related to the UPR [123]. Furthermore, the envelope protein’s ion channel (EIC) activity demonstrated to contribute to the induction of the ER stress response at late stages of IBV-infection, thus modulating different cellular responses, such as apoptosis and inflammation. EIC also induces virulence in vivo, as indicated by an experiment conducted in embryonated chicken eggs [124]. The activation of the UPR mechanism by the IBV and its proteins is displayed in Figure 5. The constant mutability of the IBV genomic RNA requires the continuous monitoring of these mutations’ impact on the induction of the ER stress and consequently on the pathogenesis of the virus. Therefore, the study of other IBV viral factors and their implications on the UPR mechanism is required to further elucidate the role of the UPR in IBV pathogenesis.

### 3.4. Deltacoronaviruses

The fourth coronavirus genus is that of the deltacoronaviruses, which was first classified in 2012. They primarily infect avians, but they are more studied in the case of porcine infections [15]. Porcine deltacoronavirus (PDCoV) possesses the typical characteristics of the coronavirus family, with its genome being a 25.4 Kb single-stranded RNA, which encodes both structural and non-structural proteins. It is believed that PDCoV evolved due to the zoonotic transmission from avians. Its clinical characteristics are similar to those of PEDV and TGEV; thus, there is confusion in the disease diagnosis [125,126]. There is little information regarding the correlation between PDCoV infection and UPR activation. In fact, Fang et al. first demonstrated that PDCoV causes ER stress and activates all three pathways of the UPR and their respective executioners in LLC-PK1 and IPI-2I cells. They found that the viral replication is enhanced by the ATF6 activation but is suppressed by the PERK-eIF2a axis. IRE1 does not contribute to PDCoV replication [127] (Figure 5). However, until now, it has not been elucidated as to which cellular processes are affected by PERK, IRE1, and ATF6 activation during PDCoV infection. Also, there are no available reports on how PDCoV proteins affect the UPR mechanism; thus, further research is needed to understand the interference of the viral factors with UPR elements and executioners.

## 4. Pharmacological Inhibition of UPRs Induced by Coronaviruses

There are numerous reports regarding coronaviruses regulating the UPR mechanism to enhance their replication and virulence. The understanding of how each coronavirus member manipulates the UPR mechanism for infection progression can contribute to the development of pharmacophores that target ATF6, IRE1, or PERK and could be used as “anti-coronavirus” drugs [128]. In this way, targeting the conserved mechanism of the UPR instead of the virus itself may reduce the chances of virus evolving into mutants that can hijack the developed therapeutic approaches.

### 4.1. PERK Pathway Inhibition

There are many small-molecular inhibitors that can target UPR transducers that have been found to have applications in contradicting SARS-CoV-2-induced UPRs. For example, the treatment with GSK2606414—a specific inhibitor of PERK—ameliorated both the PERK-induced apoptosis and inflammatory response in ORF3a-mRNA-transfected cells [107]. Furthermore, the administration of AMG PERK 4—another PERK inhibitor—resulted in a significant delay and even prevention of MERS-CoV replication both in cell culture and transgenic mice models. In particular, mice infected with MERS-CoV showed shorter recovery times and higher survival rates compared to untreated ones when they were treated with AMG PERK 44 [129]. Similarly, Chu et al. found that PERK inhibition can ameliorate MERS-CoV pathogenesis and favor the survival rate. The treatment of Huh7-infected cells with either GSK2656157 or AMG PERK 44 ameliorated the PERK-induced apoptosis. Furthermore, the group of infected hDDP4 transgenic mice, in which GSK2656157 was administered, reported suppressed apoptosis and viral replication [130]. Contrary to the antiviral role of the PERK inhibition in the case of MERS-CoV and SARS-CoV-2 infection, the GSK2606414 treatment of N2A cells infected with Porcine Hemagglutinating Encephalomyelitis Virus (PHEV)—a highly neurotropic coronavirus—resulted in the reduced phosphorylation of PERK and eIF2a and increased PHEV N protein expression. However, the treatment with salubrinal, an inhibitor of eIF2α phosphatase enzymes, showed a decline in the viral replication in a dose-dependent manner, indicating the importance of eIF2a phosphorylation [131].

### 4.2. ATF6 Pathway Inhibition

ATF6 is a one of the three main transducers of UPR, and it is reported that its signaling pathway is activated during coronavirus infection. The treatment with Ceapin-A7, a selective blocker of ATF6 signaling, reduced the PHEV virus titer in the N2A cell culture [131]. Nitazoxanide is a protein disulfide isomerase (PDI) inhibitor, playing a crucial role in maintaining a protective UPR response against the overexpression of PDIs due to ATF6 signaling activation during the coronaviruses infection. Also, nitazoxanide can induce ATF4 upregulation, which results in Nrf2 expression, a factor that protects cells against oxidative damage and thus against viral infection [132]. So, nitazoxanide could possibly be tested as a UPR regulating agent in coronavirus infections to stop disease progression. 

### 4.3. IRE1 Pathway Inhibition

IRE1 can also be pharmacologically manipulated to regulate the UPR and thus CoV infections. In a recent report, cannabidiol (CBD) and its metabolite 7-OH-CBD were found to suppress SARS-CoV-2 replication at the early stages of infection as both inhibit spike expression in infected cells. The CBD treatment of infected mice inhibited viral replication in their lungs. A possible mechanism of CBD action is by activating IRE1 and recruiting its RNase activity to probably degrade the viral RNA and to induce a physiological interferon response [133]. Another way to target IRE1 and its executioners is by treating the cells with thiopurines. In particular, the treatment of HCoV-OC43 HEK293T-infected cells with 6-thioguanine (6-TG) suppresses the strong activation of IRE1 and its executioners, delaying the UPR and thus resulting in a reduction in the viral titers [134]. Moreover, 3-ethoxy-5,6-dibromosalicyaldehyde was found to be an inhibitor of IRE1’s RNase activity and demonstrated potential antiviral effects against influenza virus. Therefore, it could also be tested in the future if it shows anti-coronavirus activity by inhibiting CoV-induced IRE1 activation [42]. Echavarría-Consuegra et al. targeted a UPR induced by SARS-CoV-2 infection, using inhibitors for each transducer or in a cocktail form. Both treatment types attenuated UPRs and significantly reduced the virion titers in the cell culture, with the cocktail of IRE1 and ATF6 inhibitors being the most promising drug combination as it resulted in a 1000-fold decrease in the release of virions [95]. 

### 4.4. ER Stress Inhibition

Apart from specific inhibitors for blocking UPR transducers’ activity, there are also pharmaceutical agents that can be used to relieve the ER stress and thus to regulate the UPR activation during a coronavirus infection. For example, YUM70, an inhibitor of Grp78, was proposed as a potential antiviral agent against SARS-CoV-2 infection. In particular, YUM70 promoted the survival of lung and liver 3D organoids infected with SARS-CoV-2. YUM70 could also inhibit the entrance of SARS-CoV-2 to cells. It downregulates the levels of spike and nucleocapsid proteins produced during infection, thus inhibiting viral replication [135]. The treatment of PEDV-infected cells with two fatty acids—docosahexaenoic acid (DHA) and eicosapentaenoic acid (EPA)—can attenuate both ER stress and PEDV replication. In particular, treatment with 100 μM of either fatty acid can relieve PEDV-induced ER stress and thus inhibit PERK phosphorylation and reduce PEDV titers [72]. During viral infections, melatonin functions as a regulator of ER stress and the UPR, manipulating the processes of apoptosis and autophagy. The antioxidant and anti-inflammatory character of melatonin combined with the promising results in treating other viral infections propose the potential supplementary administration of melatonin during COVID-19 to inhibit excessive UPR activation [136].

The occurrence of SARS-CoV-2 led to the UPR mechanism again appearing in the spotlight, thus suggesting its important role in viral replication and disease progression. As reported above, there are many recent efforts pointing to the UPR as a potential therapeutic target to develop anti-coronavirus drugs. The structural features, the mechanism of action, as well as the impact of the aforementioned inhibitors on CoV replication are summarized in Table 1. The chemical structures were created using ChemDraw® softaware (ChemDraw® Ultra, Version 8.0). However, there is little evidence regarding the in vivo administration of the existing commercially available inhibitors and even less regarding developing novel ones for treating coronavirus diseases. Also, to the authors’ knowledge, there were not any available reports regarding ongoing clinical trials of UPR inhibitors against coronavirus diseases. Therefore, these clinical and research gaps should be addressed in the future in order to achieve a more detailed insights into whether such inhibitors can be used against coronaviruses.

## 5. Conclusions

ER stress is a key component of the host response after a viral infection. The activation of the UPR is necessary to retain cellular homeostasis not only in the physiological conditions but also in diseases. The research conducted over the last decade has demonstrated that coronavirus replication leads to ER stress and initiates the UPR within infected cells. The potential of coronaviruses to infect both people and animals, along with the lack of an authorized treatment for severe cases, pose threats to public health, veterinary care, and the financial system. These factors, along with the rapid transmission, have rendered the development of new therapeutic approaches crucial. So far, there is little evidence regarding the potential use of UPR inhibitors in combating coronavirus infection, and most of the studies have focused on the in vitro testing of commercial inhibitors. Further preclinical testing and clinical trials will shed light on the potential use of UPR inhibitors as antiviral drugs. Due to the conserved nature of UPR signaling, it is important to investigate novel UPR inhibitors and their potential use as antiviral drugs. Also, limited data have been reported concerning the exact mechanism by which the virus activates the UPR and further triggers cellular responses such as redox homeostasis. Some questions that should be answered regard the possible antagonism of coronaviral proteins with BiPs upon binding to UPR transducers, the disruption of protein homeostasis, and the implications regarding pathological conditions like Ca^2+^ efflux and ROS accumulation. Further research regarding their interaction with the innate immune system is needed to clarify the virus pathogenesis. In this context, the investigation of the crosstalk between chronic UPR induction and long COVID symptoms could be addressed. 

## Figures and Tables

**Table 1:** Structural features and mechanisms of action of small-molecular inhibitors that target UPR elements.

Inhibitor Name	Structure	Action	Result	Ref
GSK2606414	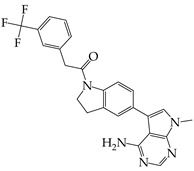	Inhibits PERK phosphorylation	Inhibits apoptosis and inflammatory response in ORF3a-transfected cells	[107]
			Enhances PHEV replication	[131]
AMG PERK 44	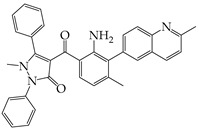	Inhibits PERK phosphorylation	Inhibits MERS-CoV replication and apoptosis	[129,130]
GSK2656157	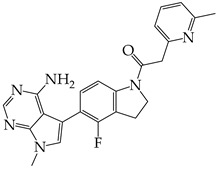	Inhibits PERK phosphorylation	Inhibits MERS-CoV replication and apoptosis	[130]
Salubrinal	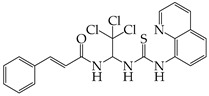	Inhibits eIF2a phosphatase enzymes	Inhibits PHEV replication	[131]
Ceapin-A7	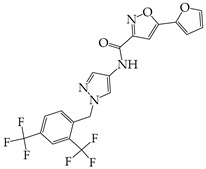	Inhibits ATF6 signaling	Reduces PHEV replication	[131]
Nitazoxanide	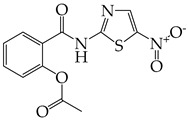	Inhibits Protein Disulfide Isomerases (PDIs)	Regulates ATF6 signaling during coronavirus infection	[132]
Cannabidiol (CBD)	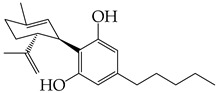	Activates IRE1 RNase activity	Inhibits SARS-CoV-2 replication	[133]
6-thioguanine	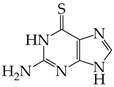	Activation of IRE1	Reduces HCoV-OC43 replication	[134]
YUM70	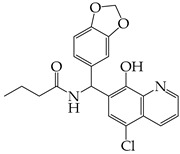	Inhibits GRP78	Blocks SARS-CoV-2 entry and reduces replication rate	[135]
Docosahexaenoic acid (DHA)	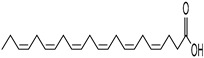	Relieves ER stress	Inhibits PERK phosphorylation and PEDV replication	[72]
Eicosapentaenoic acid (EPA)	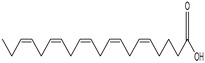	Relieves ER stress	Inhibits PERK phosphorylation and PEDV replication	[72]
Melatonin	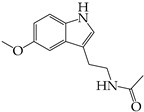	Antioxidant and anti-inflammatory	Relieves ER stress	[136]

## Data Availability

Not applicable.
